# Coping Skills, Hospitalizations, and Hopefulness in Youths with Sickle Cell Disease Treated in a Regional Outpatient Comprehensive Pediatric Center

**DOI:** 10.3390/children13050637

**Published:** 2026-05-02

**Authors:** Theodore A. Petti, Paulette Forbes, Richard Drachtman

**Affiliations:** 1 Department of Psychiatry, Rutgers Robert Wood Johnson Medical School, New Brunswick, NJ 08901, USA; 2 Bristol Myers-Squibb Children’s Hospital, Rutgers Cancer Institute of New Jersey, New Brunswick, NJ 08901, USA; forbesp2@cinj.rutgers.edu (P.F.); drachtri@cinj.rutgers.edu (R.D.)

**Keywords:** sickle cell disease, adolescents, coping, sickle cell crisis, hope, chronic illness

## Abstract

Background/Objectives: Sickle cell disease (SCD) is the most prevalent inherited pediatric hematologic disease. Pain is the most common complaint and primary reason for emergency care. Effective coping is critical to improved quality of life for individuals with SCD and other chronic illnesses. Hope, engendered by provision of comprehensive care, may explain the positive impact of effective coping and improved health outcomes. The relevance of effective coping skills and hope’s impact on repeated hospitalizations and/or length of hospitalization stay (LOS) among adolescents with SCD is considered. A regional, comprehensive pediatric sickle cell center (RCPSCC) provided the services. Methods: Patients with SCD, ages 13 through 21 years seen in a university RCPSCC (URCPCC-SCD), completed surveys: a general scale providing a broad range of positive and maladaptive coping-related issues, and KIDCOPE, a standardized scale measuring pediatric coping strategies. Medical records were reviewed for frequency of hospitalization and length of stay (LOS) for the eight months before study entry. Results: Thirty-four URCPCC-SCD outpatients, mean/median age of 16 years, entered the study, and data were analyzed for 33. All reported some sense of future hopefulness, and almost half reported feeling “tense or wound up” most of the time. Use of avoidant or negative coping strategies in response to daily stress correlated positively with increased LOS. Conclusions: Youths with SCD require effective coping strategies to improve self-efficacy and related hope for brighter futures. Individualized, comprehensive treatment and support to families and individuals at risk for sickle cell crisis are uniquely offered in a URCPCC-SCD. Their contributions to service delivery and clinical outcome are expected to enhance hope, mitigate prolonged hospitalizations, and improve adherence to treatment (N = 268).

## 1. Introduction

Sickle cell disease (SCD), the most prevalent inherited disease of childhood [1], is found primarily in individuals of African, Mediterranean, Middle Eastern, Indian, Caribbean, and Hispanic ancestry. Worldwide, approximately 300,000 infants are born annually with sickle cell anemia [2]. Over 20% of Middle Eastern individuals carry a genetic variant for either SCD or thalassemia [3]. By 2050, there is estimated to be a 30% increase in the number of individuals with the most severe and prevalent form of illness [4].

Complications of SCD are many, varied, and lead to significant morbidity and mortality. These include increased risk of life-threatening infections, pulmonary, renal, cardiac, neurological, and vascular dysfunction, life-threatening anemia, and bone and joint problems [3,5,6]. Neurocognitive deficits can result from silent and observable cerebral infarcts, significant deficits in verbal reasoning, executive functioning, and lower full-scale IQ [7]. Life expectancy is more than 20 years shorter for those with SCD than the average expected [8].

Pain initiated by obstruction of small blood vessels with sickled red blood cells is the most common SCD complaint and reason for emergency medical care [9,10]. Pain exacerbation, diagnosed as vasoocclusive (sickle cell) crisis (SCC), ranges from mild to severe debilitating episodes. They may require prolonged hospitalization for intravenous narcotics. SCD results in significant limitations for children [3]. It leads to school absenteeism, poorer school performance and participation, and lower performance on standardized tests compared to siblings and peers. Individual children with SCD manifest different levels of quality of life (QOL) that can be affected by multiple variables [3,9,11]. Studies report the wide range of adaptive and maladaptive coping strategies employed by youth to address their SCD illness [4,6,12,13,14,15,16,17,18].

Coping with the demands of chronic childhood illnesses for purposes of this discussion is defined as the intentional mental or physical action carried out to reduce the effects of stress by an internal state or external circumstances. It involves consistently employed cognitive, behavioral, and emotional strategies across contexts [3,19]. Coping styles or strategies utilized depend upon available resources, i.e., social skills, social support, problem-solving skills, health and energy levels, personal and family resources, religion, and positive beliefs. Critically, this directly relates to how the resources are mobilized, integrated, and used. Several classifications of coping strategies have been proposed and reviewed [19,20,21]. Personal and family coping skills help deal with the stress of sickle cell disease [11]. Parents’ active coping suggestions are positively associated with children’s levels of hope. Parents, on the other hand, who utilized a low level of cognitive restructuring as a coping strategy to address the pain, had children who used high levels of avoidance coping [11].

Hope is generally defined as desiring an outcome accompanied by expectation or belief in its fulfillment. It helps to explain coping strategies’ related outcomes, and subsequent perceived QOL. As a dispositional trait, hope reflects cross-situational appraisals of a person’s goal-related, relatively enduring capacities to move toward or attain those goals. It ranges from optimism, i.e., an expectation of good things happening, to hopelessness. Hopelessness entails negative expectations about the future and an accompanying sense of helplessness to affect change. Higher levels of measured adaptive behavior are associated with higher levels of hope and better QOL. Lower adaptive coping behavior to pain was associated with poorer QOL [21].

The personal adjustment and resilience of adolescents with SCD shapes their appraisal and stress coping strategies. Improved resilience and adaptive behavior may be affected by interventions to support improved adherence to recommendations for medical management of SCD [22]. Hope provides a theoretical framework to guide interventions promoting resilience in chronic illnesses [23]. It is expected to arise from a rational, practical treatment plan strengthened by adherence to evidence-based interventions. Associated strategies for improving perceived self-efficacy also play such a role [15].

Our study intended to (1) determine whether youth with poorer coping skills to manage age-appropriate stress or sickle cell pain are more likely to need repeated hospitalizations for a SCC compared to those with better coping skills, (2) gather data on likely coping strategies they would use during a SCC, and (3) give medical students the opportunity to work with child psychiatrists caring for this patient population.

Prompted by the unexpected finding of hopefulness reported by every patient, the literature review was broadened to consider hope as highly relevant to the care of pediatric patients with chronic illnesses. The implications of the findings and recommendations that follow became a focus of this report. The constructive role played by a university regional comprehensive pediatric sickle cell center (URCPCC-SCD) was expected. The overwhelming prevalence of hope in such a devastating illness was not.

## 2. Materials and Methods

Study subjects were youth receiving care in the largest New Jersey URCPCC-SCD. It provides multidisciplinary SCD care from infancy through young adulthood. The Rutgers Institutional Review Board approved the study for human use protection. Inclusion criteria were limited to youth aged 13–21 years receiving care in the URCPCC-SCD and agreeing to participate in the study. The age range was chosen based on clinical staff recommendations and studies finding SCC worsens with higher age [24]. The exclusion criterion was the inability to give informed assent/consent due to cognitive or intellectual deficits.

The study was explained and consent was obtained from the patients and their caretakers. KIDCOPE [25] was selected as the best available, easily administered, widely used measure of coping skills [26,27,28,29,30].

Its eleven items comprise representative pediatric coping strategies by children aged 13 to 18 years to common stressors for teens. For this study, they ranged from being grounded by parents for not cleaning their room to experiencing a SCC that caused them to miss an important event. Each patient completed the questionnaire. Items were rated on frequency of coping skills’ use and usefulness in the situation rated from 0 (none) to 3 (often). Based solely on the investigators’ experience with this particular SCD population, Items 1, 5, 7a, 8, and 10 were designated as negative/avoidance coping strategies for SCD. Likewise, positive coping strategies were designated for items 3, 6, 7b, and 9. No designation was assigned to KIDCOPE items 2 and 4. Additionally, Teenage Coping Strategies in Response to Conflict or Stress (TACRCS), a more general, one-page coping scale [31], was administered to provide a measure of generally employed coping strategies and sense of hopefulness. Medical records were reviewed for number of episodes requiring hospitalization and hospital days (LOS) for SCC care for the 8 months before study entry. This duration was considered sufficiently long to provide a representative sample of the dependent variables. The duration of study entry was 3 months, to reflect the medical student’s time commitment to successfully complete data collection for the project.

Non-parametric correlations of scale scores were calculated for each outcome variable, e.g., number of SCC hospitalizations and LOS. Statistical analysis was done with the JMP 10 program from SAS institute, Cary, NC, USA, specifically Student’s *t*-test and Chi-squared distribution for KIDCOPE.

## 3. Results

As summarized in Table 1, 34 patients were entered into the study, 24 males and 10 females, with mean and median ages of 16 years. Data from one male patient were considered unreliable and not considered in the analysis or detailed in the tables. The mean and median ages were 16 years. Males were slightly younger, and females had slightly fewer hospitalizations (mean 0.8 vs. 0.87) and LOS (mean 3.3 vs. 3.41, respectively).

KIDCOPE data demonstrated no significant differences between impact on hospital admissions and LOS for positive or negative coping strategies, as defined for this study. Table 2 documents that those meeting pre-selected negative/avoidant coping criteria for daily stress (N = 13) had a mean of 1.31 for hospital admissions and a median of 2.0, while the mean for the other, non-negative group was 0.53 and a median of zero. The scores for LOS days differed similarly: mean LOS of 5.15 and median of 1.0 for the negative/avoidant group versus 2.41 and zero, respectively, for the comparison group. 

To the SCD stress, contrary to our expectations, the KIDCOPE lower negative group mean for hospitalizations of 0.59 was lower than the non-negative coping group at 1.11. Similar findings were found for LOS means (2.7 vs. 3.14, respectively). However, the median was, as expected, higher in the negative/avoidant group (1.0) for stays and LOS versus the comparison group at zero for both, as demonstrated in Table 3.

Table 4 compares hospital stays and LOS by combining daily stress and SCC with scores of 14 or greater for negative coping versus those with scores of 13 or fewer with the associated number of hospitalizations and LOS with count of strategies used. As expected, the mean and median numbers for the negative coping group of strategies were greater than the comparison group, as were the number of strategies used.

The range of coping strategies for daily stress employed in our clinic sample, as detailed by TACRCS, resembles those used by adolescent medicine clinic patients in an earlier study employing that scale; however, deliberate self-harm was far less than that prompted by the inpatient adolescent psychiatric inpatient or adolescent medicine groups [31]. All patients responding to the TACRCS, as in the earlier study [31], indicated some sense of hopefulness about the future (4 somewhat and 29 very hopeful about the future). Almost half (14/31) reported feeling “tense or wound up” most of the time, 12 some of the time, and 5 hardly ever or never. To unwind, only 1 reported using medication or a pill, 5 by walking, 7 by lying down or talking, 8 by playing a game, and 17 each listening to music or watching TV. Further, 14 reported using 1 or fewer strategies, 16 used 2 or 3, and 2 used 4 or 5 strategies

## 4. Discussion

This is an exploratory and preliminary study of youth receiving outpatient psychiatric services in a regional comprehensive program. It intended to investigate the feasibility of assessing coping strategies to inform future efforts and improve positive outcomes and quality of life. We expected to identify opportunities to improve care through evidence-based assessment and interventions. Measuring the number of hospitalizations and LOS in the study provided objective measures, as contrasted to reporting subjective values. These include anxiety, depression, and quality of life that are frequently considered as outcome measures. The unanimous presence of hope in this SCD population, to our knowledge, has not been reported elsewhere. Hope can critically impact care delivery but may depend on the system within which it is delivered. URCPCC-SCDs are funded to provide comprehensive, multidisciplinary care for developing, identifying, and implementing strategies to cope with SCD and SCC. They are ideal for encouraging and maintaining hope.

The major findings of the study are the following. First, identifying and determining the best coping strategies that can be used by youth with SCD is difficult. Their selection is perhaps most dependent on circumstances. Second, the hopefulness reported by all study outpatients with SCD is likely related to their receipt of comprehensive, evidence-based care. It may be a critical factor to seek and monitor. Third, greater advocacy should be given for expansion of regional comprehensive care programs for youth with SCD and other chronic pediatric illnesses. The remainder of the discussion is meant to explain the study’s findings.

### 4.1. Assessing Coping Strategies and Their Impact

The study began with the assumption that understanding how stress is managed by youth is necessary to improve SCD clinical care. It was assumed that improving maladaptive coping strategies to everyday stress experienced by youth with SCD would reduce incidence and LOS of hospitalizations for SCCs. Scales identified to measure coping strategies for pediatric stress response were found to have significant limitations in their content, psychometrics, and populations studied. These include limited terms to classify coping strategies [20]. These pertain to the broad categorization into approach/problem-focused/control-oriented coping, as contrasted to avoidant/disengagement/escape-oriented/emotion-focused coping. Other schemas compare dispositional coping (stable coping style over time) to situational coping (behaviors used in certain circumstances) [20]. Recent theoretical models have emphasized a flexible, dynamic, context-dependent model. These match situational demands from a range of strategies as more meaningfully indicating adaptive functioning [27].

KIDCOPE was chosen for its wide and international use to assess coping strategies in dispositional and situational stressful events. It does not distinguish between adaptive and maladaptive coping. Its validity and generalizability have been questioned [27,28]. This leads to the pragmatic concept that differing types of coping strategies are needed for different illnesses and different situations. We chose to focus on negative coping styles given their greater association with poor outcomes and our imperative to reduce maladaptive behavior and emotional distress [28,32,33,34,35,36,37,38].

Multivariate factor analyses of studies across populations from different countries predominantly report two KIDCOPE factors: active/positive coping and passive/avoidant/negative coping [27,32,36]. Negative/avoidant (escape) coping has been associated with less adaptive outcomes as responses to acute and chronic stress or illness, including greater severity of depressive, ADHD, and anxiety symptoms, and a risk factor for stress symptoms and poor mental health [35]. Relevant recent insight has been gained from studies of COVID-19-related stress from consequences over time of prolonged exposure to social disruptions like those experienced with SCD. Foster et al. reported that youth in such circumstances initially use positive adaptive strategies. However, as consequences of the stress and their consequences become more challenging and accumulate over time, stresses become difficult to manage. To cope, the youth may gradually resort to maladaptive strategies and potentially exacerbate mental health problems [35]. This particularly occurs in youth with limited perceived social support and physical exercise and a dysfunctional family as delayed or persistent problems [34].

A study was conducted among Chinese junior high and high school students during the COVID-19 pandemic for trauma-related distress. It reported that negative coping for them was a risk factor for depression, anxiety, stress symptoms, and trauma-related distress (*p* < 0.05). Mental health was affected in more than one-fifth of the students due to COVID-19 stress [33]. Similar findings emanate from KIDCOPE administered to a cohort of French adolescents initiating hemodialysis. Negative coping was negatively associated with body image and relationships with medical staff. Avoidant coping was negatively associated with psychological well-being, energy vitality, and body image. In contrast, active coping was associated positively with energy vitality, quality of life, school performance, and relationships with parents and teachers [28,32]. Populations, cultures, and disciplines differ in approaches to varying degrees from each other in measuring coping strategies and their efficacy [39,40,41].

Most studies demonstrate that positive, active coping strategies result in overall benefits, while negative, avoidant coping strategies result in poor outcomes. Ernestus et al. questioned KIDCOPE and other coping scales’ validity and generalizability. They argued that such scales are at best population- or sample-specific based upon a study of over 2000 children from military families. A few studies indicate that negative/avoidant styles may be more effective in youth but do exist [27,38]. This is particularly relevant in low-resource, high-stress environments where circumstances allow individuals little control of the stressor. Cherewick et al. provided a robust discussion on the related perspectives concerning these important issues [27].

The differentiation in outcomes between the KIDCOPE designated negative strategies group, compared to the comparison group in our data analysis, did not fit comfortably into any schema. Noting that coping strategies’ valences were based solely on our clinical judgement related to SCD, non-significant differences are found in Table 2 of hospitalizations and LOS between the group designated predominantly negative strategies to a daily stress compared to the rest of the sample. But Table 3 data suggest the opposite. The contemporary view that positive/active coping strategies may be the most effective coping strategies in pediatric SCD populations may be mistaken. The best category may be selected as multiple context-dependent strategies to meet situational demands. Thus, the most effective strategies, positive, negative, or neutral, depend upon the situation. KIDCOPE may be a tool to use in that assessment and in monitoring of care base upon the perceived effectiveness. Our sample was insufficiently powered to conduct that analysis.

### 4.2. Providing Care for Chronic Pediatric Illnesses and the Role of Hope

SCD and other chronic pediatric illnesses demonstrate significant biopsychosocial complexity and require commensurate care. Providing individualized, multidisciplinary care to patients and their families provided in a URCPCC-SCD, as compared to that routinely provided (see Table 5), allows emphasizing effective coping strategies for dealing with SCD-related stresses. The multidisciplinary team approach provides the milieu and the staff to better assess and coordinate services on an individual level.

**Figure 1:**
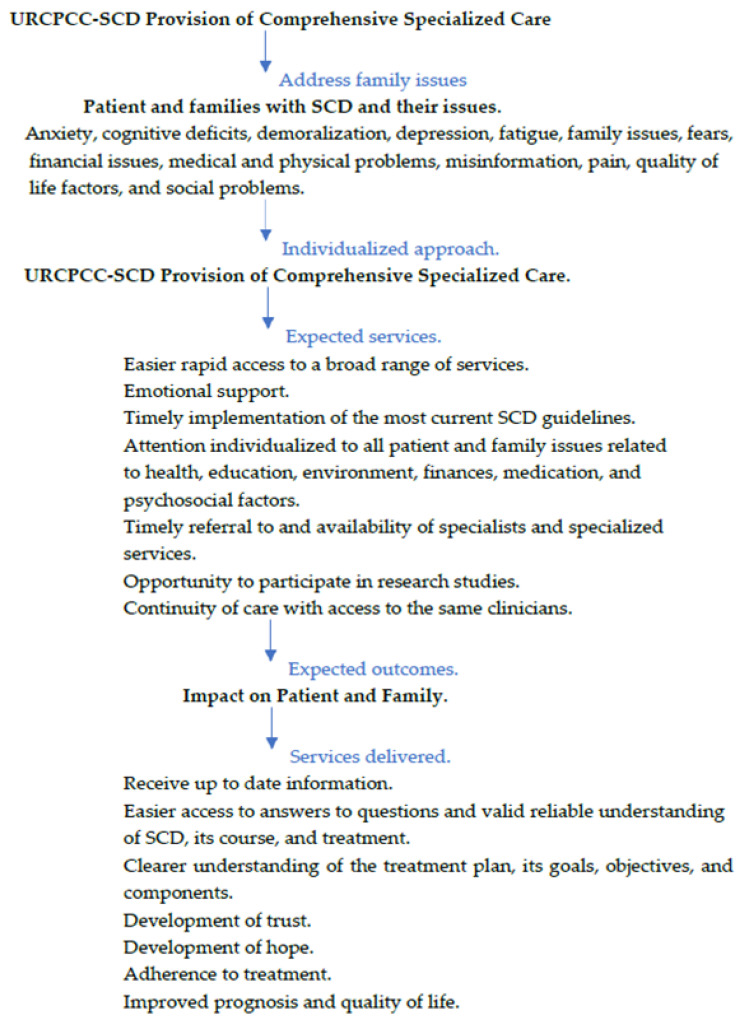
Expected course for development of hope and adherence to treatment.

Figure 1 depicts our understanding of this structure. Factors promoting hope and improved QOL and other positive outcomes are better able to facilitate the progression to trust and development of hope in mitigating factors that can be expected in the patient and families of those with SCD diagnoses. These include anxiety, cognitive deficits, misunderstanding, inadequate knowledge, depression, and fatigue. In addition, they may experience issues related to finances, health insurance, housing, transportation, work/school absenteeism due to illness, prejudices, and other related and unrelated medical and physical problems. All these issues negatively impact determinants of health and quality of life. The family-centered, multidisciplinary approach that is standard of care in URCPCC comprises social workers, hematologists, nurses with specialized training, psychologists, child life specialists, dieticians, and pediatric medical technicians.

The collaborative strategy of the diverse disciplines allows the development of individualized care plans that confront and mitigate negative outcomes that can result from fragmented care. Individualized attention is given to all patient and family issues concerning health and medication, the environment, finances, education, and psychosocial factors. Additionally, the comprehensive collaborative paradigm allows for timely referral to specialized services and specialists, as well as participation in research studies. A manifold impact on patients and their families is extensive and follows from such service delivery from updated information and easier access to answers to questions the patient and family may have about the course of SCD illness and its treatment. It facilitates development by all involved of the goals, objectives, and components of the treatment plan. From this comes trust and then hope. These then are expected to increase adherence to treatment, improved prognosis, and QOL and improved health outcomes. The breadth and depth of the URCPCC-SCD team are expected to provide greater opportunities to carry out that process, in contrast to treatment as usual. Figure 1 tracks expectations of services and outcomes by providing comprehensive specialized care.

### 4.3. Development of Hope Is a Critical, Expected Outcome Resulting from Comprehensive Care

Helplessness and hopelessness can easily result from SCD-related morbidity and mortality: diminished QOL, and accompanying barriers to developmentally expected cognitive, social, and emotional development. Outpatient youth unanimously reporting hopefulness during a clinic visit is heartening, especially since almost half (14/31; 45%) simultaneously reported feeling tense or wound up most of the time. Anxiety can be devastating without support and guidance, as advocated in practice guidelines and provided in a URCPCC-SCD. Hope’s direct and indirect effects may serve to decisively reduce anxiety for children/adolescents with SCD. This is documented for pediatric patients with cancer in efforts to improve health-related QOL [42]. Hope involves both energy and determination to meet such goals. Pain relief and strategies to accomplish those goals [11] are provided by a well-understood, comprehensive treatment plan based upon adequate resources for implementation and monitoring.

A study of 120 undergraduate students found differences between high- and low-hope subjects in feelings about their goals and related perceptions and feelings. High-hope subjects were able to estimate a greater probability of success and perceived goals set for family relations, and less difficult intimate relationships. They were able to focus greater attention on the consequences of success than those scoring at medium and low hope levels. Hope’s relationship to mental health symptoms appears stronger than to physical health symptoms and does not moderate between life stress and health symptoms [43]. Through success or outcome expectancy, hope mediates engagement in actions beyond what the perception of self-efficacy might produce. Success expectancy mediates behavioral intention and self-efficacy [44,45].

This is done through health promotion messages of positive coping strategies by URCPCC-SCD clinicians or parents by focusing on increasing perceptions of success. In turn, this more effectively induces health behaviors that promote self-efficacy. Parenting interventions to adapt their communication and modeling to their child’s needs are urged to help children learn to manage SCD. They are expected to function autonomously throughout childhood and into adulthood [46,47]. The extent of hopefulness expressed by our cross-sectional sample can be explained in many ways. One is by parents coaching and modeling active coping mechanisms, and with and by the structure and process of the services received by the youth.

Figure 1 lists many of the factors that a comprehensive program can provide to support parents. This framework directly and indirectly in turn supports the patient with SCD. It includes all the issues a recent qualitative study identified of three overarching themes facing youth with the chronic pain of SCD [48]. A condensed version of the first theme is individualized care by addressing the experience and its management of chronic sickle cell pain. This broadly includes stresses of school absences, peer socialization, hospitalizations, and autonomy development and expectations with age. Next is the impact on family and parent functioning. This involves stress experienced by parents, guilt feelings by parents for SCD inheritance, protection from pain’s affect, and need for community support. Equally important are preferences for treatment and interventions with trust by treating providers. Their expertise is high on the list to acquire coping strategies for a full, valued life. The basic desire is for providers to appreciate the youth’s pain experiences as shaped by the surrounding context of dynamic, complex experiences unique to the patient and family. Then, it is important for providers to address those unique needs and experiences by adapting interventions to optimize the child’s health, enhance behavioral pain interventions, and effectively engage in treatment [48]. Usual clinical care can rarely meet those expectations, but comprehensive programs have demonstrated their ability to do so [49]. To our knowledge, this is the first report to document the absence of hopelessness among SCD youth. These youths came from widely diverse parent socioeconomic and academic environments, reflecting the New Jersey population its URCSCC-SCD serves. Comprehensive sickle cell centers (CSCCs), established by the National Sickle Cell Anemia Control Act of 1972 and the expanded Sickle Cell Treatment Act of 2004, set standards to provide care for diverse SCD populations [50]. CSCCs utilize a family-centered, multidisciplinary model of care delivery for patients with SCD. This holistic team approach provides timely access to specialty care, as well as consistent utilization of clinical guidelines. Fiscal constraints render such a level of care out of reach in other clinical settings [51].

Adherence to prescribed treatment in managing chronic pediatric illness is problematic. It is mitigated when trust is established, and insight into the illness and its treatment is developed. The frequent SCD-related ER visits, hospitalizations, and related LOS associated with low adherence to swallowing hydroxyurea are avoided, as are associated worsening pain, fatigue, social isolation, physical function mobility, depression, and HRQOL domain scores. Such findings are consistent with earlier SCD and chronic pediatric disease studies [52]. Yet the simple task of prescribing hydroxyurea is not being implemented consistently in community practice [53]. Factors positively impacting adherence are more likely available in CSCCs and other comprehensive clinics that serve pediatric patients with chronic diseases. They include openness/transparency regarding potential benefits and potential harm and related barriers to care, the extent to which demands are manageable or not, and critically, a sense of trust to engage in the process. Rationale for this level of care is provided by a qualitative study examining factors that impact decision-making processes of caregivers and youths with SCD. Though focused on favorable factors enhancing enrollment in clinical trial research, the findings further support our study’s findings that meeting patient and family needs predisposes to hopefulness [54].

CSCCs are uniquely structured and resourced to address the basic needs of families that can assist or interfere with SCD management by providing practical, relevant informational material and meeting emotional needs. The multiple competing demands confronting patients and caregivers are elegantly outlined in a qualitative study into the bidirectional processes linking SCD management between meeting medical needs versus basic needs and financial hardships [55]. Food insecurity, which may occur in about 20% to 40% of SCD youth, as well as energy insecurity and/or housing instability, represent prime examples of such [56,57]. Such routinely addressed issues by CSCCs, and other comprehensive programs, are unlikely to be considered in routine specialty clinical or office care. SCD-related premature childhood deaths decreased 70% in the United States after CSCC funding [58]. This further supports the critical need for CSCC funding and expansion.

A multidimensional approach is needed for optimal management of painful SCC. A focus on coping with pain strategies, as we discuss, represents the bulk of psychological efforts given pain’s pervasive, persistent, and disabling presence [59]. The adaptation and effective use of coping strategies can confer added benefits for improved quality of life. Many positive/active psychosocial interventions for pain control have limited empirical support [60,61,62,63]. A Cochrane review concluded that cognitive behavioral therapy (CBT), i.e., revising one’s thoughts and actions after testing them, and relaxation by itself effectively reduce the intensity of pain immediately after treatment in SCD and other chronic pain disorders [61]. The use of the internet has been introduced as an additional resource by one comprehensive program [64]. Another comprehensive program has begun a more multicomponent, integrative program for chronic SCD pain management. It has strong preliminary efficacy to improve pain, and involves adaptations, relevance, access, engagement satisfaction, and a sense of belonging for individuals and families experiencing chronic SCD pain [49].

The severe pain accompanying SCC and the chronic pain sometimes associated with SCD are complex and demand such a biopsychosocial perspective. This is especially critical when considering recent insight into the role of physical pain as an independent risk factor for suicidality in adolescents [65]. Biological approaches include pharmacologic (e.g., hydroxyurea, opioids, and other non-controlled pain medications) [52,66,67] and related supportive approaches, e.g., cold packs, nerve stimulation, massage, acupuncture, biofeedback, relaxation, and vibration are commonly employed, but beyond this paper’s purview. Newer approaches, including gene therapy (exagamglogene autotemcel and lovotibeglogene autotemcel), prescribed L-glutamine, crizuluzimab, and antioxidant supplementation, are promising [66,67]. Gene therapy, with its great promise, is limited by cost, delivery efficiency, cancer risk, and unknown long-term effectiveness [68].

The role of parents in modeling positive or negative coping behavior and providing support for their offspring with SCD is considered critical [39,69,70,71]. Adolescent SCD coping styles often mirror those used by their parents. Individuals with parents demonstrating elevated negative thinking and passive adherence were less active, had more pain, were more distressed, and used more health services than individuals who were more positive in their thinking, more active during painful episodes, and had more appropriate responses to crises [69]. Likewise, an increased sense of helplessness was reported by parents of youth with moderate or greater depression compared to those with mild to minimal depression [70]. Parent attitudes and behaviors impact the use and range of coping skills employed by adolescents with SCD disease [71].

Awareness of effective coping strategies and their use are antidotes to perceived helplessness. They represent the foundation of hopefulness. An integrative review succinctly summarizes themes regarding hope’s role for adolescents with a chronic illness: promoting health, facilitating adjustment and coping, improves self-esteem, serves as a QOL enhancer, affects maturation, a factor in resilience, and as essential in illness and purpose of life. It is critically important for healthcare providers and researchers to focus on developing hope-inspiring strategies and the attribute of hope, rather than focusing on hopelessness and reduction of depression and other mental health symptoms. Higher levels of hope encourage and facilitate seeking out increased routes to a goal, better management of psychological distress, and use of setbacks as challenges [72].

Clinicians should inform, teach, and involve parents in any psychological or biological intervention [19,66] to foster hope and improve adherence to treatment. Moreover, as early as 1995, parental modeling and coaching were demonstrated as effective in SCD adolescents’ use of active coping strategies and their level of hope [11]. However, progress in translating evidence-based interventions in many CSCCs and elsewhere is far from universal, particularly in areas where SCD is most prevalent [58]. The fact that recommendations in the care of SCD in youth are poorly implemented is of concern. The CDC reports that the recommended ultrasound stroke screening for all SCD youth was received by less than half of them. Likewise, only half used hydroxyurea for those aged 10–16 years and even less for those aged 2–9 years. The reason was due in large part to providers’ low prescription rates with conclusions of provider biases. These can be addressed by improving their provider attitudes and knowledge toward SCD and hydroxyurea [8,73]. This is an unlikely problem for CSCCs.

The extant literature supports the importance of comprehensive assessment and individualized interventions with patients and their families that are mostly available in CSCCs. Improved funding for treatment, surveillance, and outcome evaluations is critical to fulfill the intended goals of the Federal legislation. Unanimous hopefulness may be additional support for CSCCs. However, comparison of typical clinic or office care with the single question we used, or use of a standardized hopefulness scale, e.g., the child or adult hope scales [74,75], must be done.

Analysis of the KIDCOPE data from our study arguably suggests but does not confirm that greater use of negative/avoidant coping strategies, as defined by the study for daily stress and SCC stress, are associated with more hospital admissions and LOS. Documented medians for hospital admissions and LOS are associated with greater use of negative/avoidant coping strategies for daily stress and combined daily and SCC stressors. However, the means for both variables were lower during the SCC stressor situation. These findings remain to be explained since earlier studies indicated adherence to treatment and use of positive/active coping strategies result in better outcomes.

Recent literature demonstrates and argues for comprehensive care for the SCD population to reduce morbidity and mortality in SCD [48,49,76,77,78,79]. Barriers to access SCD-specific treatment in order to improve QOL and outcomes are great where comprehensive care is not available. This typical situation results in failure to provide basic recommended clinical services. To mitigate the severity of painful crises, emphasis should be for individualized treatment planning, regardless of the service delivery system. Identifying and addressing family needs and providing coping strategies are priorities in this process. CSCCs have the structure and processes to involve families, patients, and providers through education and support [65,66]. Sessions involving the team and family utilizing a comprehensive team approach and quality metrics, as listed in Figure 1 and described above, are expected to deal with differing perceptions and needs. Examples are sharing such information as prognosis, fears, and worries [44,79,80]. The comprehensive model of care can be expected to foster a greater sense of trust, hopefulness, and subsequent increases in adherence to treatment and improved QOL and health outcomes.

As progress in understanding the psychological functions affected by SCD and its treatment, the importance of a multiprofessional team becomes more apparent. From determining the role of executive functioning, followed by the impact of SCD on processing speed, has come prescribing memantine. This drug is expected to improve working memory, attention, visuospatial and graphomotor speed, and possibly enhance executive control [81,82], all of which should improve QOL. Studies such as this with reminders to clinicians about the basics of care for SCD can encourage clinicians to improve their practice. Following guidelines for laboratory and other measures, individualizing treatment, keeping patients and parents updated about current information, and engaging in the treatment planning are practices to improve patient care and outcomes.

### 4.4. Limitations

Study limitations may help explain the contradictions in findings. Monitored factors and patient numbers (33) were limited and less than ideal for hypothesis testing. They include use of a convenience sample and self-report scales. The absence of additional objective standardized measures (e.g., hemoglobin F elevation, hydroxyurea levels, incidence of acute chest syndrome, emergency room utilization, and days with pain) is acknowledged. Unreliable reporting, e.g., a social desirability measure to address the need to look good to please the study or clinic staff, and treatment adherence are limiting factors. The scales employed are not specific for SCD and psychometrics have not been demonstrated for the TACRCS. Reliance on a single item to assess hopefulness is an additional concern. Though they ranged from two to four factors, study-generated criteria for the KIDCOPE were used to define the negative factor. Moreover, state-dependent bias is possible with a failure to consider symptom status and season at the time the scales were administered.

Data were from one regional American CSCC program that may not generalize to other countries, or comprehensive or community clinics. Our study differs significantly from international studies that provide usual or comprehensive care. Populations, cultures, and disciplines differ in approaches to varying degrees from each other in measuring coping strategies and their efficacy. A large Nigerian sickle cell center study utilized a multifaceted self-report measure. It found that diversional activities were the most commonly used, as were pain-relieving medications, talking to friends about the pain, hypnosis, prayers, and guided imagery. The most effective in pain relief strategies were prayer, relaxation exercises, and use of pain-relieving medication [41]. A small (N = 12) Brazilian study of children with SCD, ages 8 to 12 years, assessed pain coping strategies using a computerized tool. Rumination, problem solving, and cognitive restructuring were the most frequently used as the children sought positive thoughts [40]. The principles described can be applied in other settings, but not to the extent of a CSCC program.

The study was self-limiting, meant to generate hypotheses, and perhaps misdirected in its focus on negative/avoidant or passive coping strategies [48]. Failure to enroll a larger number of subjects resulted in insufficient numbers to allow conduct of more sophisticated analyses. The literature presented focuses on studies that partially support our findings and recommendations. High-quality contrary findings were not readily found to predict a more positive outcome than use of active coping skills, or that comprehensive care for chronic illness is not cost-effective. We also failed to differentiate those with and without chronic pain [40,48].

Selection of poor versus good coping skills in KIDCOPE for our data analysis was arbitrary for youth with chronic illnesses, including SCD. The effectiveness ratings of KIDCOPE items were not considered. Monitoring hopefulness for prognostic significance may not be of relevant importance. Only further research in this area can determine the relevance and role of hopefulness and coping strategies in pediatric patients with SCD and other pediatric chronic illnesses. Measuring and assessing the value or valence of coping strategies was difficult in this study. Delivery of comprehensive care, as described, may be beyond the ability of many countries to afford but certainly worth having as a goal. Future research would be expected to address these and other raised issues.

## 5. Conclusions

SCD significantly disrupts the life of youth with this illness and results in the need for patients and their families to cope with the consequences of this genetic disease. Use of effective coping skills appears to represent an important and necessary part of living a full life with this chronic illness. Future efforts to study coping strategies and their dimensional structure must account for the specifics related to the population, situational and sociocultural demands, and stressor profiles. The finding that youths with SCD receiving comprehensive, individualized services in a regional CSCC were unanimously hopeful to some extent and mostly very hopeful about their future warrants further, broader study.

Our findings suggest but do not confirm that avoiding negative/avoidant/passive coping strategies during intervals between SSCs may reduce hospitalizations and lengths of stay for SCCs. The value of the KIDCOPE to inform the care of youth with SCD is unclear. CSCCs provide services that should increase use of effective strategies to achieve those objectives with development of hope as a possible facilitator. Scientific advances, such as gene therapy and newer medications, are expected to mitigate some of the pain and suffering from SCCs and SCD. Considerations meanwhile must be directed to practical preventive and evidence-based interventive strategies to lessen the negative consequences of SCD.

Systematic efforts are required to communicate and encourage effective coping strategies for the youth and their families to enhance hope and strengthen self-efficacy. Social determinants impacting pediatric illnesses must be considered. The level of service by CSCCs is unlikely to be delivered in standard hematology care where basic recommendations are currently being inadequately implemented. CSCCs and other comprehensive care models should be well-positioned to achieve the goals outlined in the legislation funding them. In these settings, care must include psychosocial team members to devise individualized coping strategies to use during and between SCCs. The effective use of evidence-based interventions is expected to decrease hospitalizations, shorten LOS, and improve QOL. Society and its policymakers must understand the issues of chronic pediatric illness and fund accordingly.

## Figures and Tables

**Table 1:** Basic data.

	Male	Female	Total
Number	3.0 **	10.0	33.0
Age Mean *	15.70	17.20	16.00
Median *	16.00	16.50	16.00
Hospitalizations	20.00	8.00	28.00
Mean	0.87	0.80	0.85
Median	1.00	0.00	0.00
Hospital Days **	75.00	33.00	108.00
Mean	3.41	3.30	3.37
Median	1.00	0.00	1.00

Data are presented for total sample and by gender for age (years), hospitalizations, and hospital days. * Years. ** N = 22 for male hospital days.

**Table 2:** Comparison of subjects for daily stress on negative coping with scores of 7 or greater versus those with scores of 6 or fewer.

Hospitalizations	Total (N = 31)	Negative (N = 13)	Other (N = 18)
Total	28.00	17.00	11.00
Mean *	0.90	1.31	0.53
Median *	0.00	2.00	0.00
Hospital Days	Total (N = 30)	Negative (N = 13)	Other (N = 18)
Total	108.00	75.00	33.00
Mean *	5.76	5.15	1.83
Median	1.00	0.00	1.00

N = number of patients. Total is the number of hospital stays or LOS in days for the entire sample due to a typical daily stress. Negative = stays or LOS in days for those designated as negative. Other = stays or LOS in days for those designated positive plus those designated neither sickle cell crisis positive nor negative. * Total stays divided by the number of subjects.

**Table 3:** Comparison of subjects for sickle cell crisis with scores of 7 or greater on negative coping versus those with scores of 6 or fewer.

Hospitalizations	Total (N = 30)	Negative (N = 17)	Other (N = 13)
Total	23.00	10.00	13.00
Mean *	0.77	0.59	1.11
Median *	0.00	1.00	0.00
Hospital Days	Total (N = 29)	Negative (N = 16)	Other (N = 13)
Total	84.00	43.00	41.00
Mean *	3.65	2.70	3.16
Median	0.00	1.00	0.00

N = number of patients. Total is the number of hospital stays or LOS in days for the entire sample due to a sickle cell crisis stress. Negative = stays or LOS in days for those designated as negative. Other = stays or LOS in days for those designated positive plus those designated neither positive nor negative. * KIDCOPE total stays or LOS divided by the number of subjects.

**Table 4:** Comparison of subjects for hospital stays and days by daily stress and sickle cell crisis with scores of 14 or greater for negative coping versus those with scores of 13 or fewer for negative coping.

Hospitalizations	Total (N = 30)		Negative (N = 10)		Other (N = 20)	
	Stays	Strategies	Stays	Strategies	Stays	Strategies
Total	31	363	12.00	166	19	197.00
Mean *	1.03	12.10	1.20	16.60	0.95	9.85
Median *	0.00	15.00	1.00	16.00	0.00	10.50
Hospital Days	Total (N = 29)		Negative (N = 10)		Other (N = 19)	
	Stays	Strategies	Stays	Strategies	Stays	Strategies
Total	84.00	255.00	43.00	166.00	41.00	89.00
Mean *	2.90	3.65	4.30	16.60	2.16	4.68
Median	0.00	0.00	1.5	1.00	0.00	10

N = number of patients. Total is the number of hospital stays, LOS in days, and strategies for the entire sample due to combined typical daily and sickle cell crisis stresses. Negative = stays or LOS in days for those designated as negative. Other = stays or LOS in days for those designated positive plus those designated neither positive nor negative. * Total stays divided by the number of subjects.

**Table 5:** Resources in the care of the sickle cell disease patient and family.

Routine Care/Resources in Private Practice	Routine Care/Resources in Comprehensive Sickle Cell Center
Staff	Staff
Hematologist/Oncologist	Hematologist/Oncologist
Registered Nurse—General care	Advanced Practice Provider (NP/PA) *
Medical Technologist	Registered Nurse
	Pediatric Medical Technologist
	Hematology/Oncology Social worker
	Hematology/Oncology Clinical psychologist
	Pediatric Psychiatrist
	Occupational Therapist
	Physical Therapist
	Pain Medicine
	Child Life Specialist
	Primary Care Physician **

Generally accepted complements of staff for routine care compared to a comprehensive SCD center. Comparison of routine specialized care and resources based on location of services. * NP: Nurse Practitioner; PA: Physician Assistant. ** Pediatrician/family physician/advance practice nurse.

## Data Availability

Aggregated data may be obtained from the corresponding author (T.A.P.).

## Abbreviations

CSCCs	Comprehensive sickle cell centers
QOL	Quality of life
RBCs	Red blood cells
SCC	Sickle cell crisis
SCD	Sickle cell disease
TACRCS	Teenage Coping Strategies in Response to Conflict or Stress
URCPCC-SCD	University Sickle Cell Crisis (SCC) Regional Comprehensive Pediatric Center for Children with SCD
